# Sensitivity and specificity of a lateral flow immunoassay (LFI) in serum samples for diagnosis of melioidosis

**DOI:** 10.1093/trstmh/try099

**Published:** 2018-09-14

**Authors:** Gumphol Wongsuvan, Viriya Hantrakun, Prapit Teparrukkul, Mallika Imwong, T Eoin West, Vanaporn Wuthiekanun, Nicholas P J Day, David AuCoin, Direk Limmathurotsakul

**Affiliations:** 1 Mahidol-Oxford Tropical Medicine Research Unit, Faculty of Tropical Medicine, Mahidol University, Bangkok, Thailand; 2 Medical Department, Sunpasitthiprasong Hospital, Ubon Ratchathani, Thailand; 3 Department of Molecular Tropical Medicine and Genetics, Faculty of Tropical Medicine, Mahidol University, Bangkok, Thailand; 4 Division of Pulmonary and Critical Care Medicine, Department of Medicine, University of Washington, Seattle, WA, USA; 5 Department of Global Health, University of Washington, Seattle, WA, USA; 6 Centre for Tropical Medicine and Global Health, Nuffield Department of Medicine, University of Oxford, Headington, Oxford, UK; 7 Department of Microbiology and Immunology, University of Nevada School of Medicine, Reno, NV, USA; 8 Department of Tropical Hygiene, Faculty of Tropical Medicine, Mahidol University, Bangkok, Thailand

**Keywords:** *Burkholderia pseudomallei*, LFI, melioidosis, sensitivity, specificity

## Abstract

**Background:**

Culture is the gold standard for the diagnosis of melioidosis, an infection caused by *Burkholderia pseudomallei.* Here we evaluate a lateral flow immunoassay (LFI) to detect *B. pseudomallei* capsular polysaccharide (CPS) in serum samples.

**Methods:**

Patients with culture from any clinical specimen positive for *B. pseudomallei* were selected as cases. Patients who were blood culture positive for *Staphylococcus aureus, Escherichia coli* or *Klebsiella pneumoniae* as well as those who were malaria or dengue polymerase chain reaction assay positive were selected as controls.

**Results:**

The sensitivity of the LFI was 31.3% (60/192 case patients [95% confidence interval {CI} 24.8 to 38.3]) and the specificity was 98.8% (559/566 control patients [95% CI 97.4 to 99.5]) in serum samples.

**Conclusions:**

Although LFI may have limited sensitivity in serum, it can rapidly diagnose melioidosis in resource-limited settings.

## Introduction

Melioidosis, an infectious disease caused by *Burkholderia pseudomallei*, is estimated to account for 89 000 deaths worldwide every year.^1,2^ Patients commonly present with sepsis and the disease is difficult to diagnose and treat.^1^ Sepsis is defined as a life-threatening organ dysfunction caused by a dysregulated host response to infection and the cause of sepsis can be due to any organism, including bacterial, fungal and viral agents.^3^ Culture is the gold standard for the diagnosis of melioidosis, but microbiology laboratories are not widely available in developing tropical countries, where the disease is endemic. In addition, *B. pseudomallei* can be commonly misidentified as contaminants or other organisms, particularly if laboratory staff are unfamiliar with its colony appearance. *B. pseudomallei* is intrinsically resistant to a wide range of antibiotics; effective antibiotics recommended for the treatment of acute melioidosis include ceftazidime and carbapenems.^1^ Delays in diagnosis and treatment of melioidosis lead to poor outcomes. Currently, rapid diagnostic tests (RDTs) with high sensitivity and specificity are not available.^1^

Recently a lateral flow immunoassay (LFI) was developed to detect *B. pseudomallei* capsular polysaccharide (CPS) in clinical samples.^4,5^ Here we evaluate the sensitivity and specificity of the LFI in melioidosis patients and patients with alternative confirmed diagnoses who were enrolled into a prospective sepsis study in Thailand (NCT02217592).

## Methods

We conducted a prospective observational (non-interventional) study of community-acquired infection and sepsis in Sunpasitthiprasong Hospital, Ubon Ratchathani province, northeast Thailand.^3^ We prospectively enrolled adult patients (≥18 y of age) who were admitted with a primary diagnosis of suspected or documented infection made by the attending physician, were within 24 h of hospital admission and had at least three sepsis diagnostic criteria documented in the medical record.^3^ We excluded patients who were suspected of having hospital-acquired infections determined by the attending physician, had a hospital stay within 30 d prior to this admission or were transferred from another hospital with a total duration of hospitalization >72 h. Blood was drawn from all patients at the time of enrolment for culture and polymerase chain reaction (PCR) assays and serum samples were frozen at −80°C.

The Active Melioidosis Detect (AMD) LFI developed by InBios (Seattle, WA, USA; lot no. WJ1222) was used.^4^ Patients who were culture positive for *B. pseudomallei* from any clinical specimens were selected as cases. Patients with blood cultures positive for *Staphylococcus aureus, Escherichia coli* or *Klebsiella pneumoniae*, as well as positive malaria or dengue PCR assays, were selected as controls. Dengue and malaria was diagnosed by a nested PCR assay as described previously.^3,6^ Laboratory staff who performed LFI tests were blinded to the results of the bacterial cultures and PCR assays. Fisher’s exact test was used to compare categorical variables between groups. All analyses were performed using STATA version 14.2 (StataCorp, College Station, TX, USA).

## Results

From March 2013 to February 2017, 4989 adult patients presenting with community-acquired infection or sepsis were enrolled. A total of 193 patients were culture positive for *B. pseudomallei*, 209 were blood culture positive for *E. coli*, 38 were blood culture positive for *K. pneumoniae* and 62 were blood culture positive for *S. aureus*. Also, 126 patients were PCR positive for dengue and 153 patients were PCR positive for malaria. Three dengue patients and one *K. pneumoniae* bacteraemia patient also had at least one clinical specimen culture positive for *B. pseudomallei* and were categorized as melioidosis cases. Two malaria PCR-positive patients also were dengue PCR positive (n=1) or blood culture positive for *K. pneumonia* (n=1). Serum was not available from one culture-confirmed melioidosis case and 16 patients with other confirmed diagnoses. Therefore a total of 192 culture-confirmed melioidosis cases and 566 controls were included in the analysis.

**Figure 1.**
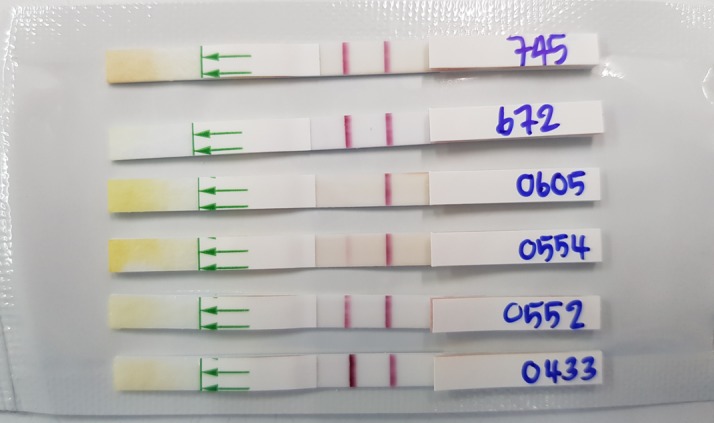
Positive, weakly positive and negative results of lateral flow immunoassay (LFI). Tests 745, 672, 0552 and 0433 show positive results, test 0554 shows a weakly positive result and test 0605 shows a negative result.

Of 758 serum samples collected within 24 h of admission, 40 were LFI positive and 27 were LFI weakly positive (Figure 1). During statistical analysis we found that the 27 weakly positive results were from 23 culture-confirmed melioidosis cases and 4 controls. Therefore all weakly positive results were considered as positive. The LFI had a sensitivity of 31.3% (60/192 case patients [95% confidence interval {CI} 24.8 to 38.3]) and a specificity of 98.8% (559/566 control patients [95% CI 97.4 to 99.5]). The sensitivity of the LFI was significantly higher in cases with *B. pseudomallei* bacteraemia than without bacteraemia (37.5% [57/153] vs 7.5% [3/40], respectively, p<0.001). The seven control samples that were LFI positive were collected from two *E. coli* bacteraemia, two dengue and three malaria patients.

## Discussion

Our findings suggest that the AMD LFI may be able to rapidly diagnose melioidosis in resource-limited settings with high specificity but limited sensitivity when testing serum samples. Affordable point-of-care RDTs are critically needed for melioidosis.^1^ The sensitivity of LFI observed is relatively lower than that of the immunofluorescence microscopic assay (IFA)^7^ (31.3% vs 48.4%, respectively). The specificity of both the LFI and IFA approaches 100%. A potential advantage of the LFI over the IFA is that the IFA cannot be used to directly test blood or serum specimens, while the LFI can be used to test whole blood, serum and other clinical specimens, including urine, sputum and pus.^4^ Further studies are needed to estimate the sensitivity and specificity of the LFI for all other clinical specimens. It is possible that the sensitivity of the LFI may increase if the LFI is used for a combination of clinical specimens (e.g., blood plus urine and other available clinical specimens) in patients presenting with community-acquired infection in melioidosis-endemic areas. The other potential advantage of the LFI is that the IFA requires immunofluorescence microscopy and trained laboratory staff while the LFI requires only a pipette and less training to use. Thus the LFI may be easier to use and relatively cheaper. The LFI can also be kept at room temperature and has a shelf life of 2 y.

The LFI could fill the current gap of RDTs for diagnosing melioidosis. A number of PCR assays for melioidosis have been developed, but none are cost effective in clinical settings.^1^ The serological diagnosis of melioidosis is difficult. The most widely used method is an indirect haemagglutination assay (IHA). However, the test is neither sensitive nor specific to diagnose melioidosis in endemic areas.^1^ Misuse of the IHA to diagnose melioidosis has led to a large number of false-positive melioidosis cases with low case fatality rates (CFRs) in the national surveillance system in Thailand.^8^ The incorrect CFRs have led to a false sense of security in the country and have limited priority setting by policymakers.^8^ A test such as the LFI, with high specificity and potentially high positive predictive value (likelihood that a patient with a positive test result actually has melioidosis), could replace the IHA to diagnose melioidosis patients in health facilities that do not have microbiology facilities, as is the case for many community hospitals in Thailand and elsewhere where melioidosis is endemic.

A limitation of this study is that the positive predictive value and negative predictive value cannot be estimated because of the case–control study design.

Our study suggests that the LFI has potential utility to diagnose melioidosis in clinical settings, particularly where resources are limited or microbiological facilities are not available. The test is highly specific but has limited sensitivity. The test could be used to diagnose patients with positive results but cannot exclude the possibility of melioidosis in patients with negative results.
